# Preconditioning of adipose tissue-derived mesenchymal stem cells with deferoxamine increases the production of pro-angiogenic, neuroprotective and anti-inflammatory factors: Potential application in the treatment of diabetic neuropathy

**DOI:** 10.1371/journal.pone.0178011

**Published:** 2017-05-19

**Authors:** Carolina Oses, Belén Olivares, Marcelo Ezquer, Cristian Acosta, Paul Bosch, Macarena Donoso, Patricio Léniz, Fernando Ezquer

**Affiliations:** 1 Centro de Medicina Regenerativa, Facultad de Medicina Clínica Alemana-Universidad del Desarrollo. Av. Las Condes, Santiago, Chile; 2 Centro de Química Médica, Facultad de Medicina Clínica Alemana-Universidad del Desarrollo. Av. Las Condes, Santiago, Chile; 3 Instituto de Histología y Embriología de Mendoza (IHEM-CONICET), Facultad de Medicina, Universidad Nacional de Cuyo, Mendoza, Argentina; 4 Facultad de Ingeniería, Universidad del Desarrollo. Av. Plaza, Santiago, Chile; 5 Unidad de Cirugía Plástica, Reparadora y Estética, Clínica Alemana. Av. Vitacura, Santiago, Chile; Medical University Innsbruck, AUSTRIA

## Abstract

Diabetic neuropathy (DN) is one of the most frequent and troublesome complications of diabetes mellitus. Evidence from diabetic animal models and diabetic patients suggests that reduced availability of neuroprotective and pro-angiogenic factors in the nerves in combination with a chronic pro-inflammatory microenvironment and high level of oxidative stress, contribute to the pathogenesis of DN. Mesenchymal stem cells (MSCs) are of great interest as therapeutic agents for regenerative purposes, since they can secrete a broad range of cytoprotective and anti-inflammatory factors. Therefore, the use of the MSC secretome may represent a promising approach for DN treatment. Recent data indicate that the paracrine potential of MSCs could be boosted by preconditioning these cells with an environmental or pharmacological stimulus, enhancing their therapeutic efficacy. In the present study, we observed that the preconditioning of human adipose tissue-derived MSCs (AD-MSCs) with 150μM or 400μM of the iron chelator deferoxamine (DFX) for 48 hours, increased the abundance of the hypoxia inducible factor 1 alpha (HIF-1α) in a concentration dependent manner, without affecting MSC morphology and survival. Activation of HIF-1α led to the up-regulation of the mRNA levels of pro-angiogenic factors like vascular endothelial growth factor alpha and angiopoietin 1. Furthermore this preconditioning increased the expression of potent neuroprotective factors, including nerve growth factor, glial cell-derived neurotrophic factor and neurotrophin-3, and cytokines with anti-inflammatory activity like IL4 and IL5. Additionally, we observed that these molecules, which could also be used as therapeutics, were also increased in the secretome of MSCs preconditioned with DFX compared to the secretome obtained from non-preconditioned cells. Moreover, DFX preconditioning significantly increased the total antioxidant capacity of the MSC secretome and they showed neuroprotective effects when evaluated in an in vitro model of DN. Altogether, our findings suggest that DFX preconditioning of AD-MSCs improves their therapeutic potential and should be considered as a potential strategy for the generation of new alternatives for DN treatment.

## Introduction

The incidence of diabetes mellitus (DM) is growing and reaching epidemic proportions with more than 260 million patients worldwide [1]. The most frequent and earliest complication associated to diabetes mellitus is diabetic neuropathy (DN), affecting 60% of type 1 and type 2 DM patients [2]. DN can affect sensory, motor or autonomic nerve fibers in any part of the body. However, the most common form is the distal symmetry sensory-motor neuropathy, affecting the lower extremities [3]. Patients afflicted with DN perceive a significant reduction in quality of life, due to the loss of sensation in different areas of the body, chronic pain and recurrent ulcerations caused by an impaired pain response, and a reduced blood flow to the affected areas [4]. These ulcerations often develop gangrenous infections leading to the amputation of the affected limb [4]. Thus, DN is responsible for 20% of the hospitalizations of DM patients and is the second leading cause of amputation worldwide, exceeded only by amputations caused by accidental trauma [5].

Although DN is clearly a multifactorial disease, the specific mechanisms that contribute to DN onset and progression are not completely understood. However, the increment of reactive oxygen species (ROS) caused by exacerbated glucose metabolism appears to be the main mechanism responsible for nerve fiber degeneration [6, 7]. Increased ROS levels induce: i) apoptosis of neurons and Schwann cells with a concomitant reduction in the local levels of several neuroprotective factors; ii) reduction in the local blood flow to nerve fibers and a decline in the angiogenic process; and iii) a chronic pro-inflammatory microenvironment [6, 7]. Therefore, reduced local levels of neuroprotective, pro-angiogenic and anti-inflammatory factors clearly correlate with the onset and progression of DN [8, 9].

Despite the continued increased incidence of DM, there is no effective clinical treatment focused on the mechanisms that lead to the development of DN. All current clinical treatments are limited to controlling blood glucose levels and to relieving symptomatic pain [10]. Therefore, more effective therapies are needed to improve nerve function in patients with DN.

Recently, it has been proposed that the use of stem cells could be a promising therapeutic strategy for the management of complex diseases like DN since unlike conventional pharmacological treatments, stem cells may act through multiple mechanisms [11]. Among different types of adult stem cells, mesenchymal stem cells (MSCs) have several comparative advantages that make them a very attractive candidate for the treatment of DN. MSCs can: i) easily be isolated from different tissues including bone marrow, umbilical cord blood and adipose tissue and can be highly expanded *in vitro* [12]; ii) exhibit cellular plasticity, so in theory they could differentiate into neurons and Schwann cells [13, 14]; iii) produce high levels of trophic cues including neuroprotective and pro-angiogenic factors [15]; iv) are able to reduce high levels of oxidative stress [16]; and v) are defined as immunomodulatory cells. Thus, by producing anti-inflammatory molecules they could reduce chronic inflammatory processes [17]. These properties have led researchers to evaluate the role of local administration of MSCs in animal models of DM as a therapeutic option for DN treatment. It has been reported that unilateral intramuscular administration of MSCs in diabetic animals induces a partial recovery of motor and sensory nerve conduction velocity, a restoration of normal structure of nerve fibers, and an increased blood flow to damaged nerves only in the transplanted side compared to the vehicle treated side [18–20]. Furthermore, authors reported an increased in the levels of several trophic factors, including beta-fibroblast growth factor (bFGF), vascular endothelial growth factor (VEGF), nerve growth factor (NGF), and neurotrophin-3 (NT3) in the periphery of nerves [18–20]. These data, added to the lack of differentiation of MSCs into neurons or Schwann cells, suggest that the therapeutic effect observed is mainly related to the secretion of paracrine molecules by MSCs, which enables the recovery of normal levels of angiogenic and neurotrophic factors [18–20].

It is well known that once in the damaged tissue, MSCs are able to sense the microenvironment and change the pattern of secreted biomolecules according to the special needs of the damaged tissue [21]. Recently, it has been reported that this response could also be achieved *in vitro* by subjecting MSCs to a preconditioning stimulus, leading to the modulation of their paracrine action [22]. The preconditioning process generally consists of incubating these cells with a toxic agent at a sub-lethal concentration. This process leads to the up-regulation of expression and secretion of several molecules that are needed to reduce damage. In this sense, it has been reported that preconditioning of MSCs with hypoxia or with reactive oxygen species (ROS) inducing agents stimulates the secretion of high levels of pro-angiogenic and anti-apoptotic factors [23–25]. Moreover, lipopolysaccharide or interferon gamma preconditioning induces the secretion of molecules with anti-inflammatory activity [26, 27]. Accordingly, this kind of strategy could be used to induce the production and secretion of an appropriate combination and ratio of trophic factors specific for a determined pathology, thus enhancing the effectiveness of the therapy.

Our work aimed to evaluate whether the *in vitro* preconditioning of adipose tissue-derived MSCs (AD-MSCs) with the iron chelator deferoxamine (DFX), a hypoxia mimetic agent with antioxidant properties, was able to increase the production and secretion of pro-angiogenic, neuroprotective, and anti-inflammatory molecules relevant for the clinical management of DN.

## Materials and methods

### Isolation, expansion and characterization of AD-MSCs

Human AD-MSCs were isolated from freshly subcutaneous adipose tissue samples (abdominal region) obtained from liposuction aspirates of four patients undergoing cosmetic liposuction at Clínica Alemana, Santiago, Chile after obtaining written informed consent. Donors were female between 22 and 56 years of age and with a body mass index (BMI) of 25 ± 1 (Mean ± SEM). All used protocols were approved by the Ethics Committee of Facultad de Medicina Clínica Alemana-Universidad del Desarrollo.

For MSC isolation, 200ml aliquots of fat were washed twice with equal volume of phosphate-buffered saline (PBS) and cut into small pieces. Samples were digested with 3mg/ml collagenase type II (Gibco, Grand Island, NY) in PBS and incubated 30 minutes at 37°C. At the end of digestion, 10% fetal bovine serum (FBS) (Gibco, Auckland, NZ) was added to neutralize collagenase. The mixture was then centrifuged at 400 g for 10 minutes to remove floating adipocytes. Pellets were re-suspended in α-minimum essential medium (α-MEM) (Gibco, Auckland, NZ) supplemented with 10% FBS and 0.16mg/ml gentamicin (Sanderson Laboratory, Santiago, Chile). Cells were plated at a density of 7000 cells/cm^2^ and cultured at 37°C in a 5% CO_2_ atmosphere. When cells reached 90% confluency, they were detached with 0.25% trypsin, 2.65mM EDTA (Sigma-Aldrich, St. Louis, MO), centrifuged and subcultured at 7000 cells/cm^2^. After two subcultures, cells were characterized according to their adipogenic and osteogenic differentiation potential. For this, MSCs were incubated with adipogenic differentiation media consisting of α-MEM supplemented with 10% FBS, 1μM dexamethasone (Sigma-Aldrich) and 10μM rosiglitazone (Cayman Chemicals, Ann Arbor, MI) for 15 days or osteogenic differentiation media composed by α-MEM supplemented with 10% FBS, 0.1μM dexamethasone, 50μg/ml ascorbate-2-phosphate (Sigma-Aldrich) and 10mM beta-glycerol phosphate (Sigma-Aldrich) for 21 days. In both cases, medium containing differentiation stimuli was replaced every three days. To evaluate adipogenic potential, cultures were stained with Oil Red-O (Sigma-Aldrich). To evaluate osteogenic potential, cultures were fixed with 10% ethanol and stained with Alizarin Red-S (Sigma-Aldrich) as previously described [21].

Immunophenotyping was performed by flow cytometry analysis after immunostaining with monoclonal antibodies against putative human MSC markers CD29 (FITC-conjugated, clone TS2/16), CD13 (FITC-conjugated, clone WM-15), CD105 (PE-conjugated, clone SN6), CD73 (PE-conjugated, clone AD2), and CD90 (PE-conjugated, clone 5E10), or characteristic markers of other cell lineages: CD235a (APC-conjugated, clone HIR2), CD31 (APC-conjugated, clone WM-59), and CD45 (APC-conjugated, clone 2D1) as previously described [28]. All antibodies were purchased from eBioscience (San Diego, CA).

### MSC preconditioning with DFX and determination of cell viability

MSCs (passage 3) at 70% confluency were incubated for 48 hours in α-MEM without FBS and supplemented with 150μM DFX (Sigma-Aldrich), 400μM DFX or double distilled water (vehicle) as a negative control. After preconditioning, cells were trypsinized and the number of living cells was estimated with trypan blue solution dye test. Briefly, a 1:1 dilution of trypan blue solution (Sigma-Aldrich) with cell suspension was used. Then 10μl of the mixed suspension was loaded into a hemocytometer, and the number of viable cells was determined under a phase contrast microscope.

### Purification of MSC secretome

After the incubation of MSCs with DFX or vehicle, the MSC secretomes were obtained by harvesting the culture media. The media were centrifuged at 400 g for 10 minutes to remove whole cells, and concentrated 10 times (v/v) using 3 kDa cutoff filters (Millipore, Midlesex, MA). In order to completely eliminate DFX from the secretome, the concentrates were washed twice with 15ml of PBS and re-concentrated again with the same filters and frozen at -80°C until use.

### Quantification of HIF-1α levels

After preconditioning with DFX, MSCs were trypsinized and lysated with cell lysis buffer containing protease inhibitor cocktail (Thermo, Waltham, MA). HIF-1α abundance was evaluated in cell lysates using the human HIF-1α ELISA kit (Abcam, Cambridge, UK), following manufacturer`s instructions. Data were normalized per mg of protein present in each sample using the BCA protein assay kit (Thermo). Data were presented as fold change in preconditioned *versus* non-preconditioned cells.

### Quantification of mRNA levels of pro-angiogenic, neuroprotective and anti-inflammatory factors

After preconditioning with DFX, total RNA was purified using Trizol (Invitrogen, Grand Island, NY) following manufacture`s instructions. Two micrograms of total RNA were used to perform reverse transcription with MMLV reverse transcriptase (Invitrogen) and oligo dT primers. Real time PCR reactions were performed in a 10μl final volume containing: 50ng cDNA, PCR LightCycler-DNA Master SYBERGreen reaction mix (Roche, Indianapolis, IN), 3mM MgCl_2_ and 0.5μM of the primers to amplify the pro-angiogenic factors VEGFα, bFGF, Angiopoietin-1 (ANG-1), and platelet-derived growth factor (PDGF); the neuroprotective factors NGF, NT3, glial cell-derived neurotrophic factor (GDNF), brain-derived neurotrophic factor (BDNF) and ciliary neurotrophic factor (CNTF) and the anti-inflammatory factors IL4, IL5, IL10 and tumor necrosis factor stimulated gene 6 (TSG6) (Table 1), using a Light-Cycler 1.5 thermocycler (Roche). To ensure that amplicons were generated from mRNA and not from genomic DNA, controls without reverse transcriptase during the reverse transcription reaction were included. Analysis of melting curve was used to ensure that only one product was amplified during the reaction. Agarose gel electrophoresis was used to characterize amplicon sizes. Relative quantifications were performed by the ΔΔCT method [21]. The mRNA level of each target gene was normalized against the mRNA levels of the housekeeping genes elongation factor 1 alpha 1 (EEF1A1) and ribosomal protein L13A (RPL13A) of the same sample. Data were presented as fold change of gene expression in preconditioned *versus* non-preconditioned cells.

**Table 1:** Specific primers for real-time reverse transcriptase-polymerase chain reaction (RT-PCR) amplification.

Gen	Primer Foward	Primer Reverse	Amplicon size
EEF1A1	AAGGATGTTCGTCGTGGCAA	GCGCTTATTTGGCCTGGATG	113 bp
RPL13a	CGTGCGTCTGAAGCCTACAA	TGTCACTGCCTGGTACTTCC	82 bp
VEGFα	TTCCAGGAGTACCCTGATGAGATCGAG	TGATCCGCATAATCTGCATGGTGATGT	145 bp
ANG1	CTTGTGGCCCCTCCAATCTAA	GGCCCTTTGAAGTAGTGCCA	94 bp
bFGF	CCGGTCAAGGAAATACACCAGT	TATAGCTTTCTGCCCAGGTCCTGT	94 bp
PDGF	GGACTGCGTTGGTGATGTAA	GGACAAGCACCACATTTCCAG	141 bp
GDNF	TGACTTGGGTCTGGGCTATGAAAC	TCGTACGTTGTCTCAGCTGCAT	87 bp
NGF	TCCAAGTCGCTGCCTTTGCATA	ATAGGCATCCCATCAGCCTCATTG	91 bp
NT3	GAAACGCGATGTAAGGAAGCCA	GGACGTAGGTTTGGGATGTTTTGC	100 bp
BDNF	CCAGAAAGTTCGGCCCAATGAAGA	AGGCTCCAAAGGCACTTGACTA	91 bp
CNTF	TCCAAGTCGCTGCCTTTGCATA	ATAGGCATCCCATCAGCCTCATTG	91 bp
IL4	GCAGCTGATCCGATTCCTGAAA	TTCCAACGTACTCTGGTTGGCT	100 bp
IL5	ACCTTGGCACTGCTTTCTACT	CCCCTTGCACAGTTTGACTC	148 bp
IL10	TACGGCGCTGTCATCGATTT	CACTCATGGCTTTGTAGATGCCTT	111 bp
TSG-6	AGCACGGTCTGGCAAATACA	ATCCATCCAGCAGCACAGAC	138 bp

### Quantification of protein levels of pro-angiogenic, neuroprotective and anti-inflammatory factors

After preconditioning, VEGF-α, NGF, and IL4 levels were measured in the MSC secretomes using the human VEGF-α ELISA kit (eBioscience); human beta-NGF ELISA kit (RayBiotech); and human IL4 high sensitivity ELISA kit (eBioscience), respectively.

### Determination of total antioxidant capacity of MSC secretomes

Total antioxidant capacity was determined in reaction volumes of 50αl of MSC secretomes using the Antioxidant Assay kit (Cayman Chemical) following manufacturer`s instructions. Results were presented as fold change in secretomes of preconditioned *versus* non-preconditioned cells.

### Detection of DFX in MSC secretomes

Presence of DFX in the MSC secretomes was evaluated by High Performance Liquid Chromatography (HPLC) based on a modified method reported by DeFrancia et al. [29]. A VWR Hitachi LaChromElite chromatographic system equipped with autosampler, column oven adjusted to 25°C, and a UV-DAD detector adjusted to 430nm was used. In order to prevent metal interference, the entire system was previously purged with a 20mM EDTA solution (Sigma-Aldrich). Deferoxamine was evaluated in samples of the MSC secretome before the washing steps and after the first and second washing steps. For this, 50μl of each sample were first complexed with 50μl of a 3mM ferric chloride solution (Sigma-Aldrich). Samples were incubated for 5 minutes to obtain the ferroxamine form and 5μl of this solution were injected into the chromatographic system with a mobile phase consisting of 15% methanol in an aqueous solution containing 0,5% triethylamine adjusted to pH 8.3. Samples were separated on a Kromasil® C18 column (4,6 x 100 mm) with 3.5μm particle size through a flow rate of 0.8ml/min. Signals were quantified using a six point DFX standard calibration curve from 10μM to 2000μM (r^2^ 0.998).

### Isolation and culture of dorsal root ganglion (DGR) neurons

DRG neurons were harvested from Wistar rats at embryonic day 15 (E15). All procedures were approved by the Institutional Animal Care and Use Committee of the School of Medical Sciences, UNCuyo (Argentina).

Embryos at E15 were obtained from terminally anaesthetized (80mg/kg pentobarbitone i.p.) pregnant rats. A total of 8 embryos from 2 litters were used in these experiments. E15 DRG neurons were cultured following previously published protocols [30]. Briefly, DRGs from all levels were dissected and enzymatically digested (0.25% trypsin in Ca^2+^ and Mg^2+^-free HBSS for 10 minutes followed by 0.25% collagenase for 10 minutes in standard DMEM at 37°C). Enzymatic reaction was stopped by addition of DMEM supplemented with 10% FBS. The ganglia were then centrifuged for 3 minutes at 600 g, the supernatant discarded and the tissue resuspended in DMEM/F12 (Gibco) supplemented with 10% FBS. The tissue was then mechanically dissociated and plated onto round coverslips (Bellco Glass, Germany), coated with 10ng/mm^2^ poly-D-Lysine (Sigma-Aldrich) and 1ng/mm^2^ laminin (Sigma-Aldrich). Neurons were kept at 37°C and 5% CO_2_ in DMEM/F12 with 10% FBS. Cell density at plating was 2x10^5^ neurons/ml. All neurons were cultured in presence of 10ng/ml mouse NGF 7S (Alomone Labs, Israel). 24 hours after plating, culture media was replaced with N2 medium (ThermoFisher, Waltham, MA) supplemented with NGF 7S. Culture medium supplemented with NGF was replaced daily.

### Incubation with MSC secretomes and apoptosis assay

Apoptosis was determined on DGR neurons on the 3^rd^ day. For this, culture media were supplemented with: i) the secretome obtained form the preconditioning of 8x10^5^ MSCs with 150μM DFX; ii) the secretome obtained form the preconditioning of 8x10^5^ MSCs with 400μM DFX; iii) the secretome obtained form 8x10^5^ non-preconditioned MSCs or iv) the vehicle. Culture medium contains a basal 25mM glucose that is essential for DRG neuron culture. To produce a hyperglycemic insult, 20mM additional glucose (yielding a total of 45mM glucose) was added to the medium 4 hours after supplementation with the secretomes and maintained for 24 hours.

To determine the percentage of apoptotic cells, DRG neurons were fixed with 4% paraformaldehyde (Merck) and evaluated for apoptosis by the in situ TdT-mediated dUPT nick end labeling (TUNEL) assay with an in situ cell death detection kit (Promega, Madison, WI) following manufacturer’s instructions. Cells were counterstained with 4’-6’-diamidino-2-phenylindole (DAPI) (Invitrogen). Cells treated with DNAse I (Fermentas, Burlinton, Canada) were considered as positive controls. Cells treated with reaction solution but without terminal transferase were considered as negative controls. DRG neurons were examined using a Fluoview FV10i confocal microscope (Olympus, Tokyo, Japan). More than 2500 DGR neurons were examined for each condition. Data were analyzed with the Olympus FV10-ASW4.1 software. Results were presented as percentages of apoptotic cells.

### Statistical analysis

Data were presented as mean ± standart error of the mean (SEM). Multiple group comparisons were performed by analysis of variance (ANOVA) followed by Bonferroni *post hoc test*, while comparisons between two experimental groups were performed by Student’s *t* test. *p*<0.05 was considered statistically significant.

## Results

### Characterization of human AD-MSCs

**Fig 1:**
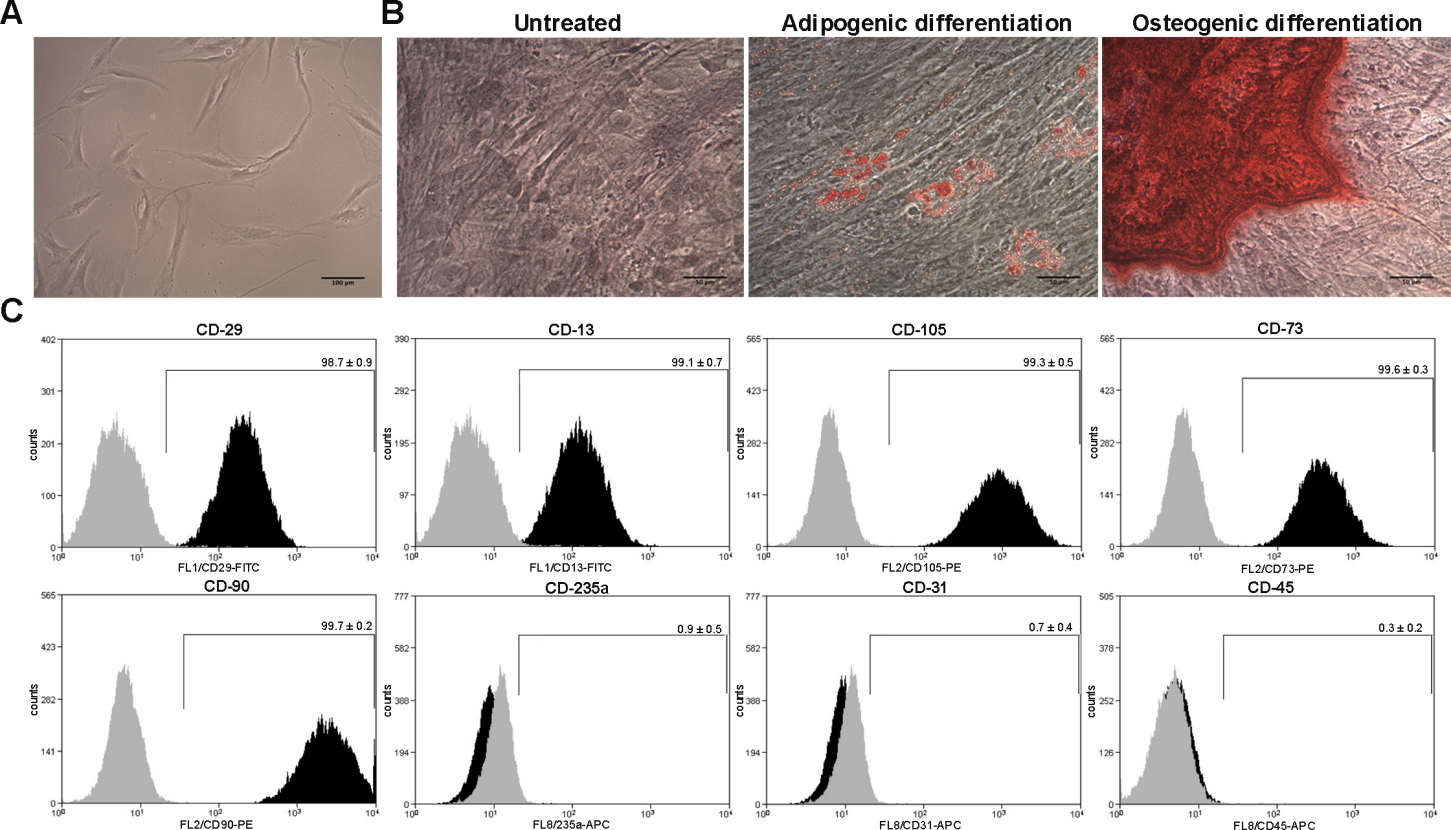
Characterization of human AD-MSCs. (A) Morphological characteristics of MSCs five days after plating. (B) Representative images of AD-MSCs at passage three, differentiated towards adipogenic and osteogenic lineages. Lipids droplets were stained with Oil red-O whereas hydroxyapatite precipitates were stained with alizarin red. (C) Flow cytometry analysis of AD-MSCs at passage three, for putative human MSC markers (CD29, CD13, CD105, CD73 and CD90) and markers characteristic of other cell lineages (CD235a, CD31 and CD45). Grey histograms represent isotype controls while black histograms represent specific antibodies. Data shown are representative of MSCs isolated from four different donors. Experiments were repeated three times at technical level.

Human AD-MSCs were isolated from subcutaneous adipose tissue samples obtained from liposuction aspirates of four female patients undergoing cosmetic liposuction. Five days after plating, MSCs demonstrated the characteristic long spindle-shaped morphology (Fig 1A), and exhibited adipogenic and osteogenic differentiation when they were stimulated with the appropriate differentiation media (Fig 1B). Additionally, cells at passage three were characterized according to the presence of putative human MSC surface markers and absence of surface markers characteristic of other cell lineages. Flow cytometry analysis showed that AD-MSCs were more than 98% positive for CD29, CD13, CD105, CD73, and CD90, and less than 1% positive for CD235a, CD31 and CD45 (Fig 1C), indicating that the isolated cells had the typical MSC phenotype.

### Deferoxamine preconditioning increases HIF-1α abundance without affecting MSC morphology and survival

**Fig 2:**
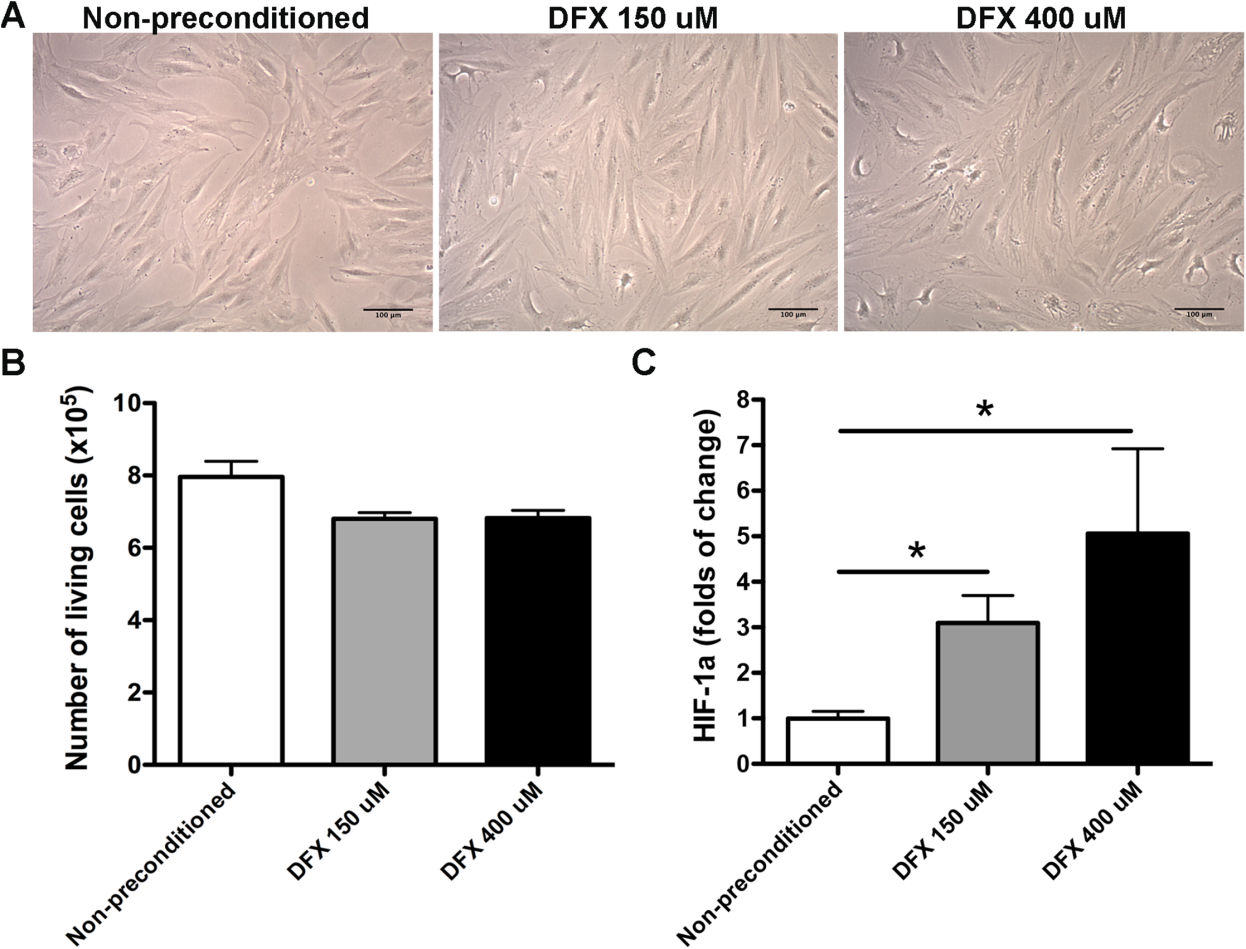
Deferoxamine preconditioning increases HIF-1α abundance without affecting MSC morphology and survival. (A) Morphological characteristics of MSCs exposed to 150μM DFX, 400μM DFX or to the vehicle for 48 hours. (B) Quantification of MSC survival determined by trypan blue exclusion assay for cells exposed to 150μM DFX, 400μM DFX or to the vehicle for 48 hours. Data is presented as number of living cells. (C) Quantification of HIF-1α protein levels in lysates of MSCs exposed to 150μM DFX, 400μM DFX or to the vehicle for 48 hours. Data were expressed as fold change in preconditioned versus non-preconditioned cells. Quantitative data correspond to mean ± SEM. N = 4 per experimental group (biological repeats). Experiments were repeated three times at technical level. * *p< 0.05*.

AD-MSCs were incubated with 150μM or 400μM of the hypoxic mimetic agent DFX or with the vehicle (non-preconditioned cells). We chose these doses of DFX since it has been previously reported that, for others stem cells types, the incubation with this dose range improves their therapeutic potential and has only a modest impact on stem cells survival [31, 32]. We observed that 48 hours of preconditioning did not induce cell morphology changes (Fig 2A) and did not significantly reduce cell survival (Fig 2B) (6.82x10^5^ ± 0.20x10^5^ cells after preconditioning with 150μM DFX; 6.83x10^5^ ± 0.22x10^5^ cells after preconditioning with 400μM DFX compared to 7.95x10^5^ ± 0.43x10^5^ cells in the non-preconditioned condition), suggesting that 48 hours of preconditioning with 150μM or 400μM of DFX have nontoxic effects on MSCs.

HIF-1α has been described as the main hypoxia marker. We observed that MSC preconditioning for 48 hours with DFX induced a dose-dependent increase in HIF-1α abundance (3.09 ± 0.60 folds of change for 150μM DFX preconditioned cells and 5.06 ± 1.86 folds of change for 400μM DFX preconditioned cells, compared to 1 ± 0.16 folds of change for non-preconditioned cells) (Fig 2C) supporting the hypoxic inductive effect of DFX.

### Deferoxamine preconditioning increases the expression and secretion of pro-angiogenic, neuroprotective and anti-inflammatory factors

It has been previously reported that high levels of HIF-1α, associated with hypoxic preconditioning, induce the expression and secretion of high levels of pro-angiogenic factors including VEGFα [33]. However, we wanted to evaluate if DFX preconditioning of AD-MSCs could also induce the expression and secretion of neuroprotective and anti-inflammatory factors relevant for the clinical management of DN.

**Fig 3:**
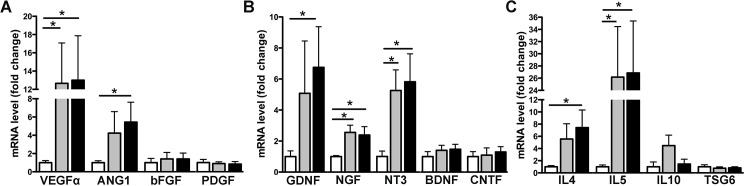
Deferoxamine preconditioning increases the expression levels of pro-angiogenic, neuroprotective and anti-inflammatory factors. Total RNA was obtained from MSCs exposed to 150μM DFX, 400μM DFX or to the vehicle for 48 hours and subjected to quantitative reverse transcriptase–PCR analysis. (A) Quantification of the mRNA levels of the pro-angiogenic factors VEGFα, ANG1, bFGF and PDGF. (B) Quantification of the mRNA levels of the neuroprotective factors GDNF, NGF, NT3, BDNF and CNTF. (C) Quantification of the mRNA levels of the anti-inflammatory factors IL4, IL5, IL10 and TSG6. Data of each target gene was normalized against the mRNA levels of the housekeeping genes EEF1A1 and RPL13A of the same sample and presented as fold change of expression in preconditioned versus non-preconditioned cells. White bars represent non-preconditioned MSCs, gray bars represent MSCs preconditioned with 150μM DFX and black bars represent MSCs preconditioned with 400μM DFX. Data is shown as mean ± SEM. N = 4 per experimental group (biological repeats). Experiments were repeated three times at technical level. * *p< 0.05.*

As expected, we observed that preconditioning of MSCs with 150μM DFX or 400μM DFX induced a significant increase in the mRNA levels of the pro-angiogenic factors VEGFα and ANG-1, determined by RT-qPCR (Fig 3A) (for VEGFα: 12.66 ± 4.42 folds of change for 150μM DFX preconditioned cells and 13.01 ± 4.87 folds of change for 400μM DFX preconditioned cells, compared to 1 ± 0.23 folds of change for non-preconditioned cells; for ANG-1: 4.24 ± 2.35 folds of change for 150μM DFX preconditioned cells and 5.44 ± 2.18 folds of change for 400μM DFX preconditioned cells, compared to 1 ± 0.21 folds of change for non-preconditioned cells). Importantly, these preconditioning also induced the expression of the neuroprotective factors GDNF, NGF and NT3 (Fig 3B) (for GDNF: 5.08 ± 3.37 folds of change for 150μM DFX preconditioned cells and 6.75 ± 2.62 folds of change for 400μM DFX preconditioned cells, compared to 1 ± 0.37 folds of change for non-preconditioned cells; for NGF: 2.56 ± 0.46 folds of change for 150μM DFX preconditioned cells and 2.39 ± 0.54 folds of change for 400μM DFX preconditioned cells, compared to 1 ± 0.06 folds of change for non-preconditioned cells; for NT3: 5.26 ± 1.33 folds of change for 150μM DFX preconditioned cells and 5.82 ± 1.80 folds of change for 400μM DFX preconditioned cells, compared to 1 ± 0.35 folds of change for non-preconditioned cells). We also observed a significant increase in the expression of the anti-inflammatory factors IL4 and IL5 (Fig 3C) (for IL4: 5.55 ± 2.50 folds of change for 150μM DFX preconditioned cells and 7.43 ± 2.89 folds of change for 400μM DFX preconditioned cells, compared to 1 ± 0.20 folds of change for non-preconditioned cells; for IL5: 26.17 ± 8.30 folds of change for 150μM DFX preconditioned cells and 26.85 ± 8.53 folds of change for 400μM DFX preconditioned cells, compared to 1 ± 0.30 folds of change for non-preconditioned cells). Increased expression of VEGF-α, ANG-1, GDNF, NGF, NT3, IL4 and IL5 after DFX preconditioning seems to be specific for the mentioned molecules, since this effect was not seen in other factors of the same families such as bFGF and PDGF (pro-angiogenic molecules), BDNF and CNTF (neuroprotective molecules), IL10 and TSG6 (anti-inflammatory molecules) (Fig 3A–3C).

**Fig 4:**
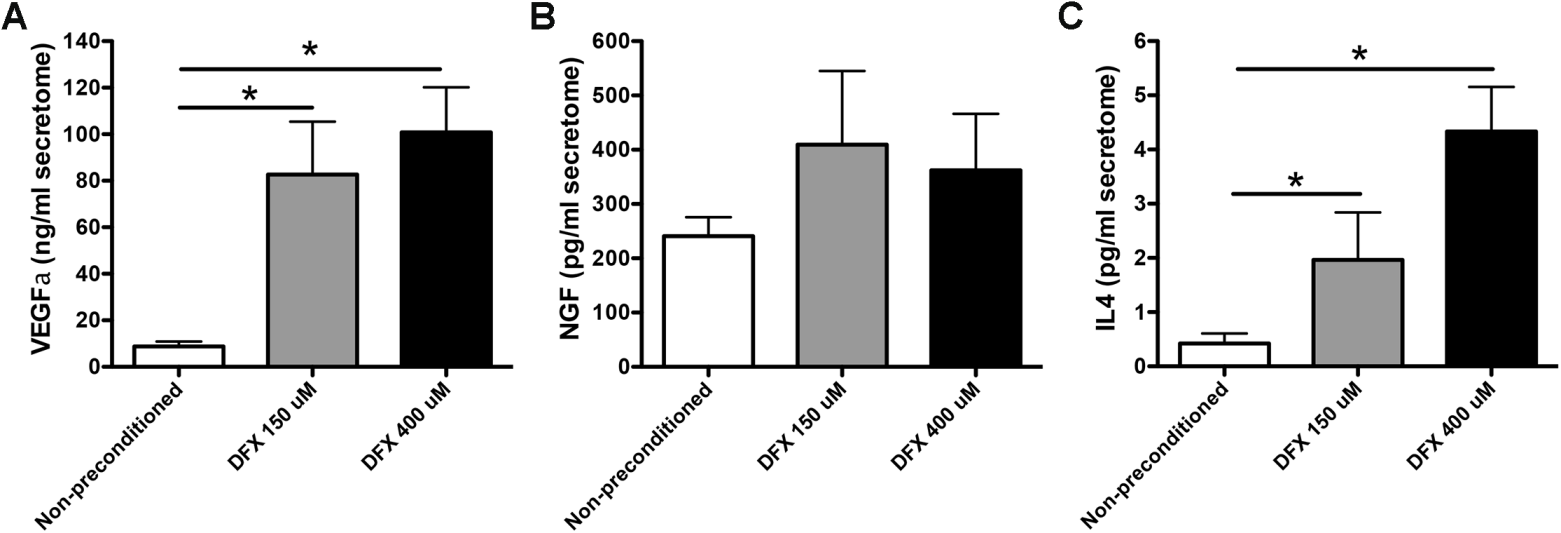
Deferoxamine preconditioning increases the secretion of pro-angiogenic, neuroprotective and anti-inflammatory factors. The secretomes obtained from MSCs exposed to 150μM DFX, 400μM DFX or to the vehicle for 48 hours were concentrated 10 times (v/v) using 3 kDa cutoff filters and the protein levels of selected factors were evaluated by ELISA. (A) Quantification of VEGFα in MSC secretomes. (B) Quantification of NGF in MSC secretomes. (C) Quantification of IL4 in MSC secretomes. Data is presented as mean ± SEM. N = 4 per experimental group (biological repeats). Experiments were repeated three times at technical level. * *p< 0.05*.

Next, we evaluated whether the increase in the mRNA levels observed after DFX preconditioning could also be correlated with an increase in the protein levels of these factors in the MSC secretome. For this, we chose a representative factor of each family and measured its protein level in the MSC secretomes by ELISA. In accordance with our RT-PCR data, we observed that preconditioning of MSCs by incubation with DFX (150μM or 400μM) induced a significant increase in the protein levels of the pro-angiogenic factor VEGFα and in the anti-inflammatory factor IL4 compared to the levels detected in the secretome of non-preconditioned MSCs (Fig 4A and 4C) (for VEGFα: 82.63 ± 22.77 ng/ml in the secretome of 150μM DFX preconditioned cells and 100.8 ± 19.35 ng/ml in the secretome of 400μM DFX preconditioned cells, compared to 8.75 ± 2.13 ng/ml in the secretome of non-preconditioned cells; for IL4: 1.96 ± 0.85 pg/ml in the secretome of 150μM DFX preconditioned cells and 4.33 ± 0.82 pg/ml in the secretome of 400μM DFX preconditioned cells, compared to 0.42 ± 0.18 pg/ml in the secretome of non-preconditioned cells). With regard to NGF and DFX, preconditioning induced an increase in the protein level of this neuroprotective factor; however, it did not reach statistical significance (Fig 4B) (409.5 ± 135.4 pg/ml in the secretome of 150μM DFX preconditioned cells and 362.1 ± 104.1 pg/ml in the secretome of 400μM DFX preconditioned cells, compared to 240.7 ± 34.8 pg/ml in the secretome of non-preconditioned cells).

### Deferoxamine preconditioning increases the antioxidant capacity of MSC secretomes

**Fig 5:**
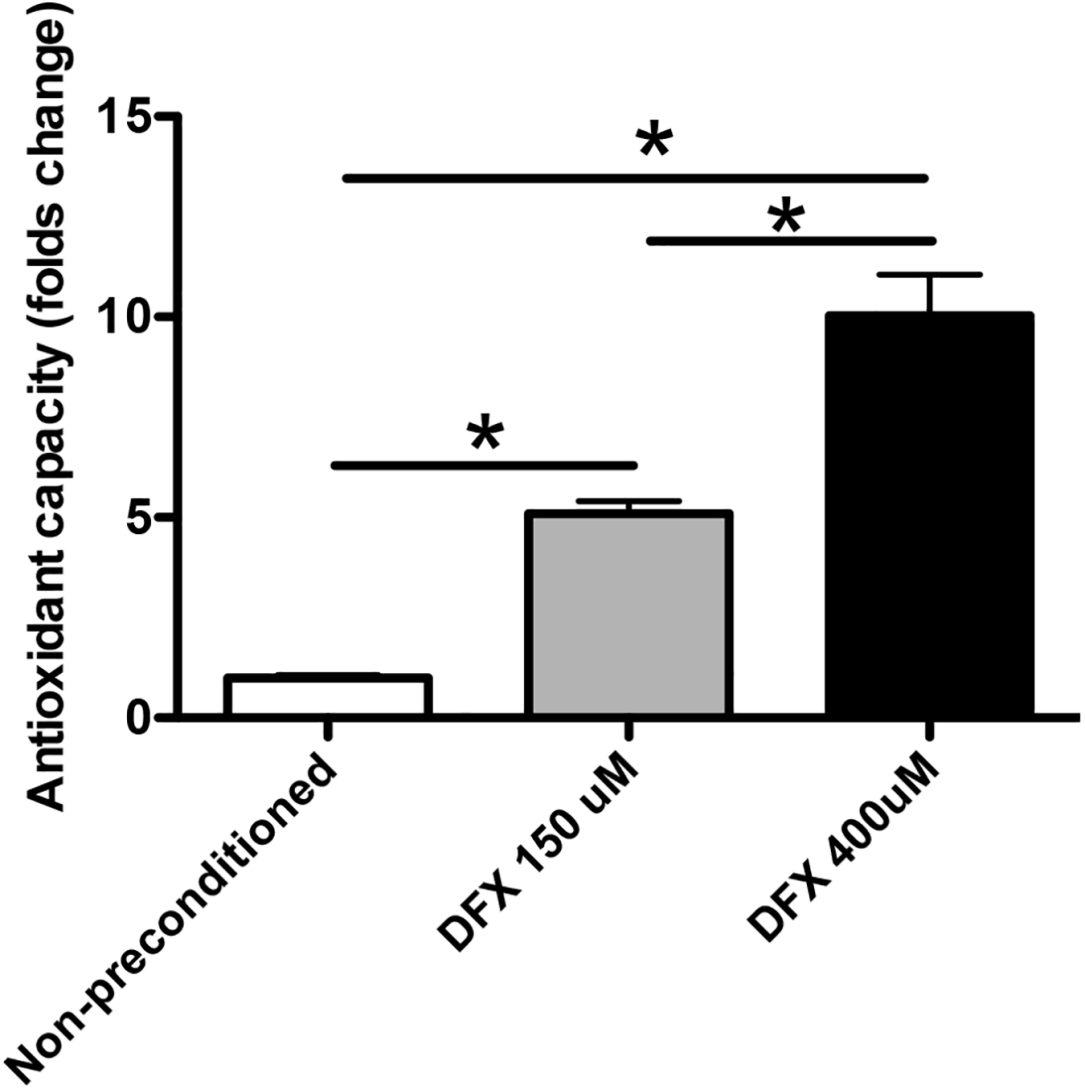
Deferoxamine preconditioning increases the antioxidant capacity of MSC secretomes. The secretomes obtained from MSCs exposed to 150μM DFX, 400μM DFX or to the vehicle for 48 hours were concentrated 10 times (v/v) using 3 kDa cutoff filters. Total antioxidant capacity was quantified on the secretomes by colorimetric reactions. Data were expressed as fold change of total antioxidant activity in preconditioned versus non-preconditioned MSC secretomes. Data correspond to mean ± SEM. N = 4 per experimental group (biological repeats). Experiments were repeated three times at technical level. * *p< 0.05*.

One of the regenerative mechanisms associated with the therapeutic effects of MSCs is the efficient handling of high levels of oxidative stress [16]. It has been proposed that some of the antioxidant molecules produced by MSCs could also be secreted [16, 34], reducing ROS levels in the surrounding microenvironment. Therefore, we evaluated whether DFX preconditioning of MSCs could increase the antioxidant capacity of the secretomes. The antioxidant system used in this study includes enzymes such as superoxide dismutase, catalase and glutathione peroxidase; macromolecules such as ferritin, and an array of small molecules including ascorbic acid, α-tocopherol, β-carotene, reduced glutathione, uric acid and bilirubin. Thus, the combined antioxidant activities of all the constituents of the MSC secretomes were assessed. We observed that DFX preconditioning significantly increased the antioxidant capacity of MSC secretomes in a concentration-dependent manner (Fig 5) (5.1 ± 0.3 folds of change in the secretome of 150μM DFX preconditioned cells and 10.1 ± 1 folds of change in the secretome of 400μM DFX preconditioned cells, compared to 1 ± 0.07 folds of change in the secretome of non-preconditioned cells).

### Deferoxamine is not present in the final secretomes obtained from preconditioned MSCs

**Fig 6:**
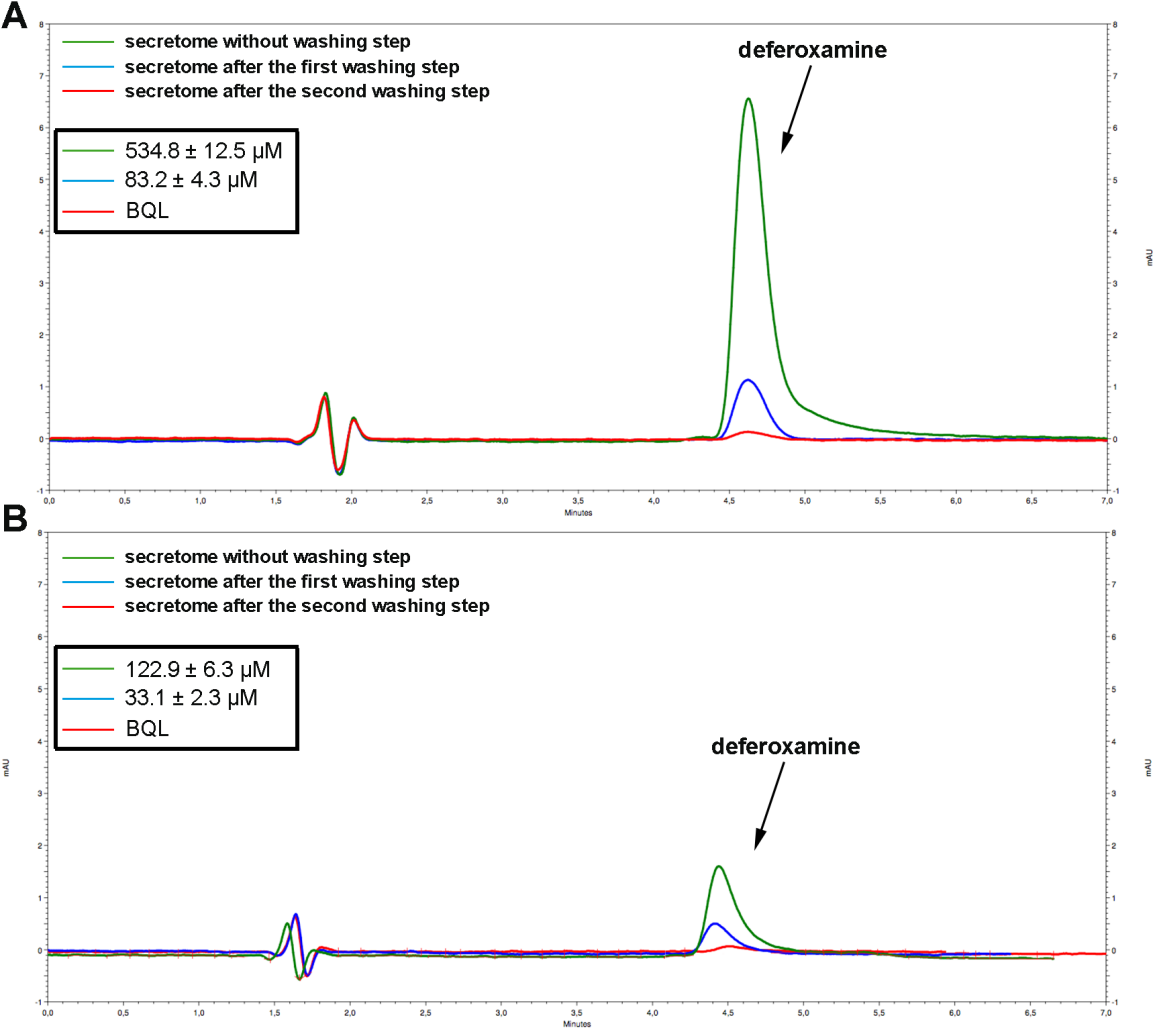
Deferoxamine is not present in the final secretomes obtained from preconditioned MSCs. The secretomes obtained from MSCs exposed to 150μM DFX or 400μM DFX for 48 hours were concentrated 10 times (v/v) using 3 kDa cutoff filters. The concentrates were washed twice with 15ml of PBS and re-concentrated again with the same filters. (A) Quantification by HPLC of DFX in the secretome of MSCs exposed to 400μM DFX before the washing steps and after the first and second washing steps. (B) Quantification by HPLC of DFX in the secretome of MSCs exposed to 150μM DFX before the washing steps and after the first and second washing steps. Quantitative data is presented as mean ± SEM. N = 4 per experimental condition (biological repeats). Experiments were repeated three times at technical level. BQL: Below quantification limit.

In order to use the MSC secretome as a therapeutic product, the preconditioning stimulus must be completely eliminated after the preconditioning process. Due to the small size of DFX, we aimed to eliminate this drug from the MSC secretomes subjecting them to two sequential washing and re-concentration steps. By HPLC, we observed that DFX was present in both (150μM and 400μM DFX) MSC secretomes before the washing steps, but its concentration was reduced to very low levels (below quantification limits) after the second washing step (Fig 6A and 6B).

### The secretomes of DFX preconditioned MSCs reduce glucose-induced DRG neuron death

Loss of DRG neurons has been described *in vivo* during DN [35] and *in vitro* after high-dose glucose-induced injury [36, 37]. Therefore, the *in vitro* stimulation of DRG neurons with diabetes-inducer conditions would allow for the screening of neuroprotective compounds with potential to prevent DN.

To evaluate the neuroprotective ability of the MSC secretomes, DRG neurons were pretreated with the secretomes of 150μM DFX preconditioned MSCs, the secretome of 400μM DFX preconditioned MSCs, the secretome of non-preconditioned MSCs or the vehicle. The dose for the secretomes was based on previous reports [38]. Four hours later, glucose level in culture media was raised from basal level (25mM glucose) to hyperglycemic level (45mM glucose). Twenty-four hours later, DRG neurons were fixed and TUNEL-stained.

**Fig 7:**
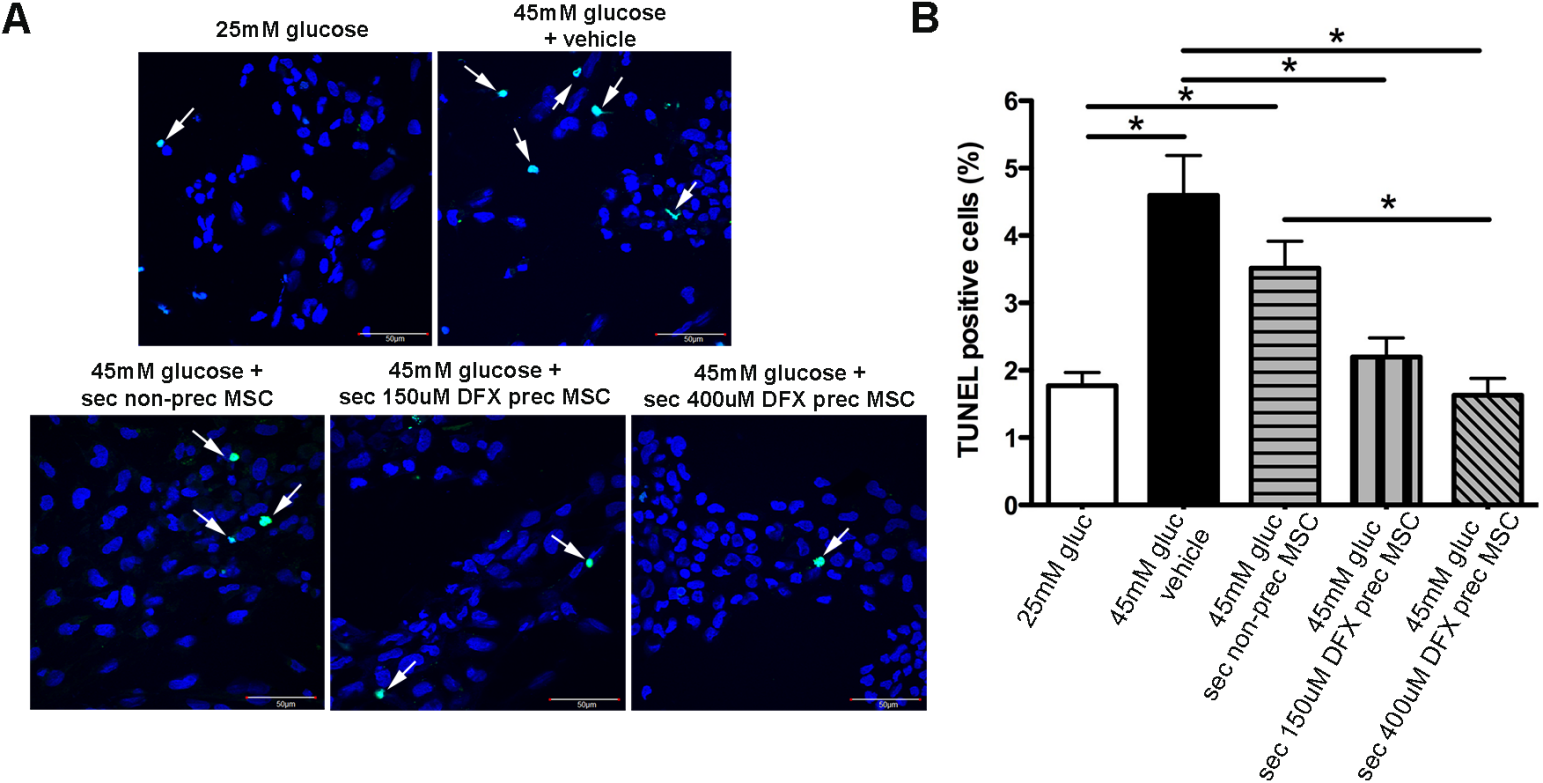
The secretomes of DFX-preconditioned MSCs reduce glucose-induced DRG neuron death. DRG neurons were pre-incubated with the secretomes of 150μM DFX-preconditioned MSCs, 400μM DFX-preconditioned MSCs or non-preconditioned MSCs for 4 hours. Hyperglycemic insult was then induced increasing glucose levels to 45mM. (A) Representative images of DRG neurons 24 hours after hyperglicemic insult. Green nuclei represent apoptotic TUNEL-stained cells (marked by arrows). Nuclei were counterstained with DAPI (blue). (B) Quantification of percentage of TUNEL-positive nuclei in each experimental condition. Quantitative data is presented as mean ± SEM. N = 4 per experimental condition.

As previously described, hyperglycemic insult induced a significant increase in the rate of apoptosis of DRG neurons (Fig 7A and 7B). (4.6 ± 0.6% in the 45mM glucose DRG neurons compared to 1.8 ± 0.2% in the 25mM glucose DRG neurons). Pre-incubation of DRG neurons with the secretome of non-preconditioned cells produced only a modest decrease in TUNEL positive cells, while the pre-incubation with the secretome of 150μM DFX and 400μM DFX preconditioned MSCs significantly decreased glucose-induced DRG neuron death (Fig 7A and 7B) (2.2 ± 0.5% in DRG neurons pre-treated with secretome from 150μM DFX preconditioned MSCs; 1.6 ± 0.3% in DRG neurons pre-treated with secretome from 400μM DFX preconditioned MSCs and 3.5 ± 0.4% in DRG neurons pre-treated with secretome from non-preconditioned MSCs).

## Discussion

Mesenchymal stem cells have emerged as a tool with great clinical potential. MSCs can be isolated from a wide range of tissues [39, 40], of which bone marrow is the traditional and most frequently used source. However, bone marrow aspiration is invasive and is accompanied with donor morbidity [41]. In contrast, adipose tissue is more accessible, can be obtained repeatedly under local anesthesia with a minimum of patient discomfort and has a higher yield of MSCs compared to that of bone marrow [42, 43]. Furthermore, AD-MSCs share many proprieties with bone marrow MSCs. Thus, adipose tissue is nowadays the preferred source of MSCs for regenerative therapy [42, 43]. Initially, it was postulated that the major mechanism associated with the therapeutic effects observed after the administration of MSCs in animal models of different diseases was related to the ability of these cells to migrate to damaged tissues, differentiate into the required cell type and replace dead cells [44]. However, it has been recently observed that the number of transplanted cells that reach and functionally integrate into damaged tissues is too low to account for their therapeutic effects [45]. Therefore, the generation of a pro-regenerative microenvironment, through the paracrine secretion of trophic factors, is now the most accepted therapeutic mechanism associated to MSCs. Thus, MSCs have been described as the “injury drugstore” of the body [46]. This phenomenon is also observed after local administration of MSCs in preclinical animal models of DN, in which it has not been possible to detect differentiation of MSCs into neurons, Schwann cells or endothelial cells despite an evident recovery of motor and sensory nerve conduction velocity, recovery of the normal structure of nerve fibers, and increased blood flow to damaged nerves [18, 19, 47]. These data suggest that different trophic factors secreted by MSCs are responsible for reducing the symptoms characteristic of DN.

The broad spectrum of molecules secreted by MSCs is known as the secretome. In humans, the MSCs secretome consists of over 100 biologically active molecules including different types of cytokines, chemokines, and growth factors [48]. In different pathologies such as acute myocardial infarction and brain or liver damage, it has been shown that the administration of the MSC secretome is able to reproduce, at least in part, the therapeutic effect observed after the administration of living cells [49, 50] while at the same time reducing the therapy complexity and the cost. However, a major issue associated with the use of MSC secretome is that in most cases the concentration of cytokines and growth factors are too low for therapeutic use [51].

Recently, it has been reported that, due to the high plasticity of MSCs, it is possible to modify the MSC secretome baseline composition, by subjecting these cells to an *in vitro* preconditioning stimulus, producing a biological product with a defined combination and ratio of biomolecules specific for a determined pathology [23–25].

In this context, many different strategies are being developed in order to boost the paracrine effect of MSCs, such as genetic engineering or pre-treatment with different physical or chemical factors. For example, it has been reported that preconditioning of MSCs with hypoxia (1%O_2_) stabilizes the HIF-1α protein and induces the secretion of high levels of pro-angiogenic factors [52], while the preconditioning of MSCs with LPS or IFN-γ induces the secretion of molecules with anti-inflammatory activity [26, 27]. In this study, the iron chelator DFX was used as a preconditioning stimulus because it acts as a hypoxic mimetic agent, interfering with the degradation of HIF-1α [53]. We observed that the *in vitro* preconditioning of human AD-MSCs for 48 hours with two concentrations of DFX did not affect MSCs viability. As expected, these treatments induced the stabilization of HIF-1α, and led to the overexpression of genes related to pro-angiogenic responses including VEGFα and ANG-1. These results are in agreement with earlier studies indicating that DFX can induce VEGFα expression in various cell types, such as pancreatic beta cells [54], endothelial cells [55], and bone marrow stromal cells [33]. However, we also observed that the preconditioning of MSCs with DFX could also increase the expression of potent neuroprotective factors including NGF, GDNF and NT-3, and cytokines with anti-inflammatory activity such as IL4 and IL5. This enhancement in the production of therapeutic molecules after DFX preconditioning was specific for the mentioned factors, since this effect was not seen for other molecules of the same families *e*.*g*. bFGF, PDGF, BDNF, IL10, and TSG6. Additionally, this study demonstrated that the up-regulation of the mRNA levels of the mentioned factors correlates with an increased secretion of these protective molecules. This data suggests that modulation of culture conditions by incubation with DFX can be used to generate tailored MSCs, as well as to improve the MSC secretomes, making them suitable for different therapeutic applications.

Finally, we observed that the secretome obtained after the preconditioning of MSCs with DFX showed increased antioxidant capacity, enhancing its possible therapeutic use. These results corroborate previous reports indicating that MSCs are able to secrete high levels of molecules with antioxidant activities and also antioxidant enzymes including superoxide dismutase, catalase, and glutathione reductase [16, 34]. This study demonstrated that DFX preconditioning could also modulate the antioxidant properties of MSCs.

The pathogenesis of diabetic neuropathy is multifactorial, involving both metabolic and vascular components [3]. However, at the molecular level, the increase in ROS levels in mitochondria due to exacerbated glucose flux has been proposed as the central player in DN pathogenesis [6, 7]. It has been reported that high intracellular ROS levels can induce DN development acting on several levels. For example, neurons and Schwann cells are especially sensitive to the damage induced by ROS, due to their high density of mitochondria, which leads to cell apoptosis [7]. By secreting a broad range of neuroprotective factors (NGF, GDNF, NT-3, BDNF and CNTF), Schwann cells play a central role in neuron survival but also in the axonal regeneration of peripheral nerves after injury [56, 57]. Therefore, the early death of these cells in DN induces a significant reduction of the local levels of neuroprotective factors generating a vicious cycle that enhances nerve fiber degeneration and prevents their regeneration [8]. Of special importance are the neurotrophins NGF and NT-3. These trophic factors are clearly involved in growth, maintenance and survival of sympathetic and sensory neurons [58, 59]. Additionally, increased ROS levels induce alterations in endothelial cells, generating loss of the vasodilation capacity of epineural blood vessels and reduction in pro-angiogenic factors production. This phenomenon leads to a reduction in the microcirculation of blood vessels that irrigate nerve fibers and the induction of ischemia and neuronal dysfunction [60, 61].

Finally, increased oxidative stress also leads to the activation of the poly ADP-ribose polymerase (PARP) pathway, which is responsible for regulating pro-inflammatory cytokine expression. This creates a chronic inflammatory state that exerts direct toxic effect on neurons and Schwann cells. This process accelerates the demyelination and atrophy of the nerve fibers and contributes to the chronic pain characteristic of DN patients [62].

In diabetic rats, there is evidence of mitochondrial dysfunction and ROS accumulation in DRG neurons, followed by induction of apoptosis [56, 63]. Additionally, it has been reported that the *in vitro* incubation of DRG neurons with high glucose induces oxidative stress, caspase cleavage and the ultrastructural hallmarks of apoptosis [37, 64]. Thus, DRG neurons are a good model for the initial screening of potential drugs for the treatment of DN [37, 64].

Using this *in vitro* model we observed that incubation of DRG neurons with the secretomes of DFX preconditioned MSCs reduced their apoptotic rate after challenge with high glucose, suggesting a therapeutic potential of the studied secretomes.

High prevalence of DN has driven the effort to develop different types of experimental therapies guided to block the mechanisms involved in its progression. For example, oral or intravenous administrations of antioxidants, mainly -lipoic acid, in different animal models of DN produce an increase in nerve conduction velocity and a reduction in chronic pain [65, 66]. Additionally, the local administration of recombinant neuroprotective, pro-angiogenic, or anti-inflammatory factors was correlated with a decreased apoptosis of neurons and Schwann cells and with an increase in both motor and sensory nerve conduction velocity [67–72].

However, while most of these therapies have shown positive results in diabetic animals, the transfer of these procedures to clinical studies has been very unsuccessful [73, 74] and none of mentioned strategies are being routinely applied in clinical practice [75]. This seems to be mainly related to the fact that DN pathogenesis is multifactorial and involves many interrelated mechanisms. Therefore, a single molecule-based therapy is unlikely to be effective.

A therapeutic approach simultaneously targeting the angiogenic, neurodegenerative, inflammatory and oxidative processes, like the administration of the secretome of MSCs subjected to a preconditioning stimulus with DFX, may have great value in DN treatment. Furthermore, this treatment could be repeated over the time, since it does not generate an immunological rejection [76].

Compared to the use of living stem cells, this type of product has several advantages including: i) higher biosafety, since the administration of the MSC secretome has less bioethical considerations, and fewer risks of tumor formation than the administration of living cells; ii) immediate availability, since the MSC secretome can be lyophilized, favoring its storage, while living cells require extensive time for expansion; iii) greater reproducibility of the therapeutic effects, since biological variability of MSC secretomes can be minimized as it has a more defined composition (which can for instance be determined by a proteomic approach) while using MSCs may have more unpredictable effects; iv) easier handling, since biodrug administration is easier, safer and requires less complex medical facilities than those necessary for the administration of living cells: and v) lower production costs, since the MSC secretome can be produced in large quantities, reutilizing the cells. Therefore, the use of the MSC secretome as a therapy would reduce the high costs that are usually associated with the production of cell therapies for which the cells must be obtained and expanded for each patient.

Compared with other preconditioning stimuli like cobalt chloride, DFX has the advantage that it has been used in humans for more than three decades to treat iron overload diseases [77]. Therefore, it is believed that the administration of this agent to humans is safe. This study demonstrated that, even when used at high dose, DFX has no toxic effect on MSCs, which permits fine adjustment of the preconditioning by varying the concentration of DFX. However, one important feature for the clinical translation of this kind of biological therapy is that the preconditioning stimulus can be completely removed from the final product. In this sense, we have demonstrated that due to its small size, after two washing and re-concentration steps, DFX concentration could be reduced to undetectable levels.

Further evaluation is needed to demonstrate whether the local administration of the secretome of MSCs preconditioned with DFX could reverse the main functional and structural alterations characteristic of DN. Nevertheless, we believe that this strategy might also be useful for the treatment of others neurodegenerative disorders including amyotrophic lateral sclerosis, Huntinton’s and Parkinson’s disease and traumatic brain injury, in which the presence of neuroprotective, pro-angiogenic and anti-inflammatory factors are relevant [78]. We suggest that the administration of the secretome of MSCs preconditioned with DFX in animal models of these diseases could stimulate neural growth, decrease apoptosis, reduce levels of free radicals, enhance angiogenesis and regulate inflammation, promoting the functional recovery of the damaged organ.

Additionally, the preconditioning of MSCs with DFX could also be used prior to the administration of the cells to enhance their therapeutic effect. For example, it has been reported that one of the main factors that limits the therapeutic efficacy of MSCs is the poor survival of transplanted cells into the ischemic target tissue, which presents high levels of inflammatory factors and free radicals generated by oxidative stress [79]. Recent studies involving overexpression of anti-oxidant, anti-apoptotic, or anti-inflammatory genes in MSCs have shown improved survival after transplantation [80, 81]. Therefore, the preconditioning of MSCs with DFX prior to their administration could also help to increase the tolerance of these cells to the hypoxic microenvironment, enhancing their survival in the damaged tissues. Furthermore, preconditioned MSCs may support the angiogenic process better than naïve MSCs because they secrete more VEGF and enhance the proliferation and migration capacity of endothelial cells [82].

Finally, experiments using autologous MSCs from diabetic patients have suggested that these cells are less potent in their therapeutic potential, due to disease-induced cell dysfunction, limiting their clinical use [83, 84]. Still, the preconditioning we report here, with the overproduction of several trophic factors, could be a way to overcome these noxious defects.

## Conclusions

Overexpression of survival molecules and growth factors appears to be a potent strategy to enable MSC therapeutics. This paper described a preconditioning strategy that led to an increase in the transcription and secretion of several potent pro-angiogenic, neuroprotective, and anti-inflammatory factors. The secretomes of preconditioned MSCs had neuroprotective effects when evaluated in an *in vitro* model of DN.

This upregulation of various classes of biologically important factors may be one of the greatest benefits of stem cell therapy compared to any single protein or gene therapy, enabling a concerted effect of multiple neuro-angiogenic and anti-inflammatory cytokines necessary for neurovascular recovery.
